# Sex and Care: The Evolutionary Psychological Explanations for Sex Differences in Formal Care Occupations

**DOI:** 10.3389/fpsyg.2019.00867

**Published:** 2019-04-17

**Authors:** Peter Kay Chai Tay, Yi Yuan Ting, Kok Yang Tan

**Affiliations:** ^1^Centre for Ageing Research and Education, Duke-NUS Medical School, Singapore, Singapore; ^2^Humanities and Social Sciences, Murdoch University, Singapore, Singapore

**Keywords:** sex differences, occupation, sexual strategies theory, sexual division of labor, care styles

## Abstract

Men and women exhibit clear differences in occupational choices. The present article elucidates sex differences in terms of formal care occupational choices and care styles based on evolutionary psychological perspectives. Broadly (1) the motivation to attain social status drives male preference for occupations that signals prestige and the desire to form interpersonal affiliation underlies female preference for occupations that involve psychosocial care for people in need; (2) ancestral sex roles leading to sexually differentiated cognitive and behavioral phenotypic profiles underlie present day sex differences in care styles where men are things-oriented, focusing on disease management while women are people-oriented, focusing on psychosocial management. The implications for healthcare and social care are discussed and recommendations for future studies are presented.

## Introduction

Sex differences are evident in formal care occupations across cultures. Regardless of sex equity, males and females appear to undertake certain care occupations more over others. For instance, nurses are overwhelmingly females (O’Connor, 2015) while physicians and healthcare managers are predominantly males (Ku, 2011; Lowe, 2011). In the current review, we use evolutionary psychological (EP) frameworks (i.e., sexual strategies theory, sexual division of labor) to elucidate the innate tendencies driving sex differences in formal care occupations. Broadly, we argue that males provide care in positions that advertise social status while females are motivated to provide care for affiliation purposes. In addition, we postulate that males tend to focus on non-human (i.e., things) aspects of care while females tend toward more human (i.e., psychosocial) aspects. Based on this analysis, we examine the implications for healthcare and social care sectors.

## Definitions of Care

In the current article, we focus on formal care in the healthcare and social care domains. *Formal care* refers to paid care and to some extent volunteered care coming from government and non-profit organizations. We exclude domestic helpers because they tend to differ from other formal carers in terms of cultural background and ethnicity; formal carers are usually employed in institutional settings as opposed to domestic helpers who are usually employed by agencies and private enterprises. We focus on formal care particularly in the healthcare and social care setting because sex differences in formal care has not been subjected to EP analysis to our knowledge.

There are clear sex preferences in the Science, Technology, Engineering, and Mathematics (STEM) domains (Lippa, 2010), and we expect the same sex preferences to underlie the formal care domains. Although sex ratio in STEM occupations has become less unbalanced in recent years, the sex differences remain in social disciplines such as Health and Welfare which has a greater proportion of female Ph.D. graduates (59%), contrasting with engineering, manufacturing, and construction (28%) (European Commission, 2016). Sex differences are also notable within formal care occupations. Globally, females outnumber males overwhelmingly and this sex difference is consistent across all ages, where the bulk of the female workers occupy people oriented professions such as nurses and social workers (Gupta et al., 2003; Rocheleau, 2017; Ministry of Manpower, 2018). The Luxemburg Income Study conducted with eighteen participating countries in Europe, America, Asia, and Oceania showed that across countries, at least 62–85% of health workers are females (Gupta et al., 2003). Specifically, a greater proportion of females worked in the nursing and midwifery specializations compared to physicians. In the following sections, we use EP theoretical frameworks to explicate the evolutionary roots that underlie these patterns.

## Evolutionary Psychological Theories on Sex Differences Related to Care

In this article, EP analysis refers to conceptual analyses based on the postulation that evolutionary-driven innate factors underlie emergent behavioral tendencies, attitudes, and preferences. Specifically, we elucidate EP causes to understand how sex differences in formal care emerge and examine the implications for these differences.

### Sexual Strategies and Occupational Choice

In humans, sexual selection involves a showcase of physical and psychological traits, evolved on the basis of attracting the opposite sex (Darwin, 1871; Puts, 2010). The sexual strategies theory explicates sex-specific qualities that confers evolutionary benefits which are thus attractive to the opposite sex: Among males, social status translates into reproductive advantage because females prefer males who possess traits which signal their ability to invest resources in their offspring (Buss, 1989; Buss and Schmitt, 2017), in part because of females’ physical vulnerability and demands associated with pregnancy and childbirth (Bjorklund and Shackelford, 1999). Thus, reproductive success among low status males is greatly reduced (Hopcroft, 2006; Fieder and Huber, 2007). This contrasts with male mate choice which largely focuses on indicators of fertility and child bearing capacities such as youth (Buss and Schmitt, 2017). Thus, female social status is less relevant to males, and females are comparatively less concerned about their own social status (Low et al., 2002).

For males, occupation provides one of the most salient opportunities to enhance their social status. Thus, males are more focused on their careers, devoting their resources to work that will generate high-impact products and gain recognition at work particularly during mid adulthood (Ferriman et al., 2009). Relating to social status, male sex role in most cultures emphasizes dominance (i.e., competitive behaviors) understood as “judgment of the status, power, and/or competence of others and their underlying motivation to seek it” (Adams et al., 2015). As such, hierarchical occupations which provide opportunities for attaining leadership positions will be particularly attractive to males.

While males are more concerned with social status and develop skills to advance their career, females tend toward affiliation and focus on building interpersonal relationships. In most societies, female sex role emphasizes on affiliation (i.e., communal behaviors) (Eagly, 1997; Eagly and Wood, 2013; Adams et al., 2015). Affiliation refers to the “judgment of solidarity, friendliness, and/or warmth and the underlying motivation to seek it” (Adams et al., 2015). In contrast to the *fight-or-flight* tendency among males, the *tend-and-befriend* hypothesis highlights that females are equipped with the natural skills to provide care, and creating and maintaining social networks (Taylor et al., 2000). For instance, an experimental study demonstrated that females are more likely to help others especially when it does not involve risks (Eckel and Grossman, 2008). Furthermore, *both* females and males prefer females as confidantes because females are more likely to engage in conversations related to relationships and provide social support (Barbee et al., 1993; Bank and Hansford, 2000; Kenrick, 2012), highlighting the relevance for females take on formal care roles that promote affiliation.

### Sexual Division of Labor and Care Styles

Sexual division of labor highlights that males and females utilize their natural abilities to procure resources and achieve cooperative mutualism, where males function as “hunters” while females function as “gatherers” (Bird and Codding, 2015). Particularly, a great part of human ancestry is spent in the Savannah environment where males take on the role of hunting for large prey and toolmaking while females take on the role of providing childcare, and gathering and planting foods (Murdock and Provost, 1973; Marlowe, 2007; Bird and Codding, 2015). Even in cases when females are involved in hunting, they typically play supportive roles such as assisting the males in their hunting trips (Hurtado et al., 1985) or tracking game (Biesele and Barclay, 2001). Given this division of labor, traits favoring the sex-specific activities will developed accordingly among males and females. In particular, because hunters travel long distances over wide territories compared to gatherers, males develop better technical skills such as spatial and navigational ability, throwing skills and focused attention (Frost, 1998; Stoet, 2011). Conversely, given that gathering requires social interaction among individuals within a small area, females developed better social and linguistic skills (Stoet, 2011).

Consistent with this notion, males and females show abilities and preference for things and people, respectively (Lippa, 1998). Meta-analytic studies revealed sex differences in terms of occupational preferences where females show preferences for people oriented jobs, while males for object oriented jobs (Su et al., 2009), and these sex-specific preferences were observed in all 53 nations of a review study where the size of the sex differences were uncorrelated with global variation in gender equity (Lippa, 2010). This indicates that sex differences in occupational preferences are universal across cultures and do not diminish in more gender egalitarian cultures. As sex differences in sex-differentiated traits and occupational preference are robust and pervade across cultures, we expect similar sex patterns for care styles. Particularly, a greater proportion of males in formal care will take on tasks that require hard, technical skills and independence, while more females occupy the “softer” or more nurturing aspects of care. We now turn to the implications on healthcare and social care settings based on the current EP arguments.

## Implications on Healthcare and Social Care Occupational Domains

### Theoretical Implications

Existing theoretical frameworks in the literature tend to explain sex differences in formal care based on socialization and sex-role theories rather than evolutionary theories. As opposed to evolutionary theories, sex-role theories emphasize the impact of societal influence based on a person’s biological sex, leading to the development of sex stereotypic traits and perceptions (Vanwesenbeeck, 2009; Eagly and Wood, 2011). For instance, sex-role theory predicts that sex stereotyping about cognitive abilities and preferences is developed through socialization and nations that are more sex equal would have lower sex differentiated occupational pursuit (e.g., Riska, 2011). Yet, the sex differences appear universal, and unrelated to sex egalitarianism (Charles and Bradley, 2009; Lippa, 2010). In particular, the pursuit of STEM occupations observed the largest differences in sex egalitarian countries (e.g., Germany, Sweden, and Switzerland), and the smallest differences were observed in the least sex egalitarian countries (e.g., Colombia, Indonesia, and Tunisia) (Charles and Bradley, 2009). Thus, EP theories may provide better explanations for sex differences in occupation choices and provide more precise predictions. For instance, EP theorists argue that males take on STEM occupations because they tend to have innate preferences and possess relevant cognitive abilities such as spatial, mathematical and mechanical competencies (Geary, 2014), suggesting that sex preferences rather than sex inequality drives unbalanced sex ratios across occupations. This line of argument is further supported by studies on homosexuals displaying sex atypical preferences (e.g., Lippa, 2005). In particular, occupational choice appears to be influenced by sexual orientation where homosexual males report stronger preferences toward jobs such as school teacher, florist and social worker, and homosexual females report stronger preferences for jobs such as builder, carpenter, and electrical engineer compared to their heterosexual counterparts; bisexuals report job preferences between heterosexuals and homosexuals (Lippa, 2008).

Theories which exclude EP explanations risk mislabeling innate preferences as inequalities in formal care occupations. Some scholars argue that sexual discrimination underlies sex differences in formal care occupations and presumed that removing it would result in sex equity across occupations. For instance, it has been assumed that the underrepresentation of female physicians can be attributed to sex discrimination (Hannawi and Al Salmi, 2018). Notwithstanding that certain types of sex discrimination such as unequal remuneration exists and should be eliminated, extant evidence suggests that eradicating sex discrimination will not lead to equal sex ratio for formal care occupations (Lippa, 2010; Stoet and Geary, 2018). Furthermore, sex inequity fails to account for drastically low male workers in formal care occupations with little sex discriminatory practices like nursing and social work. Thus, scholars attempting to explain sex differences should integrate EP frameworks to develop more holistic and accurate theories to explain sex differences in formal care occupations.

### Human Resource

Given that sex egalitarian countries tend to have the greatest sex differences in personality and occupational choices (Charles and Bradley, 2009; Lippa, 2010), sex specific policies such as increasing vacancies for the sex with lower hire proportion may not be effective. For instance, although demand for male-dominated blue-collar professions (e.g., manufacturing, mechanics) is shrinking while demand for female-dominated healthcare industry is growing, the resultant excess in male population in the work force did not lead to a corresponding increase in male employment in “pink-collar” formal care professions such as nursing or healthcare aides (Dill, 2017). Similarly, an overemphasis on sex-ratio reversal policies undermines the stronger effect of innate preferences. In particular, policies skewed toward promoting atypical sex employment may not ultimately lead to balanced sex employment and may be counterproductive. For instance, medical enrolment in favor of female applicants may place some eligible male applicants at a disadvantage (McKinstry, 2008). Furthermore, even though female students have a slight advantage in many STEM subjects compared to male students, female students nevertheless tend to pursue non-STEM education (Stoet and Geary, 2018).

Sex-role theorists argue that female physicians encounter greater occupational barriers because of the expectation that females are homemakers (Buddeberg-Fischer et al., 2010). Our present analysis suggests that instead, females have a natural inclination to provide care to their families. This understanding will change how we encourage females to remain as physicians. Particularly, females tend to trade-off their career development particularly when they have children so that they can devote more time for the family and more broadly, females also divert more resources toward the community, friends, and less on their careers (Ferriman et al., 2009). Thus, understanding innate preferences for sex differences underlying the effect of family demands and parenthood on career choices for medicine can provide potential solutions to facilitate the enrolment and maintenance of female physicians (Buddeberg-Fischer et al., 2010; Riska, 2011). On the other hand, males tend to undertake jobs that emphasize strong leadership and offer high extrinsic rewards such as higher income and prestige as indicative of one’s social status (Ku, 2011). Policies aimed to increase hiring of males in occupations such as nursing and social work will be more effective if is it coupled with changing societal perceptions of such professions. Awareness about the barriers toward females is nonetheless important, yet ignoring potential EP driven factors that would attract females and males into professions conventionally occupied by the opposite sex would be ineffective.

### Preferences and Competencies in Care Tasks

The people-thing dimension highlights that males and females possess innate abilities and preferences (Lippa, 1998), and may manifest in specific care tasks that people adopt. For instance, males and females tend to focus on different aspects of a problem (e.g., an illness) and use different methods to solve the same problem (e.g., treatment method) in formal care settings. Concurring with the notion that ancestral males are largely responsible for the manufacturing of tools and weapons (Puts, 2010), modern males tend to be technology and skills oriented, and select formal care occupations such as surgical specialties, medical technicians, paramedic, radiology, and pathology (Hojat et al., 2002, 2005; Simpson, 2007). In contrast, females are people-oriented and tend to adopt roles in caring, understanding and supportive services in diagnosis and treatment contexts (Hojat et al., 2002, 2005), and occupy human and relational based professions such as childcare workers, social workers, nurses, health aides and community/social service specialists (Rocheleau, 2017). Even male nurses tend to focus on technical competence and rationality to preserve their masculine identity (Simpson, 2007).

To support males in nursing roles, it may be fruitful to develop their technical and rational skills as part of the job scope. Given the long standing perception that nurses receive poor wages (Evans, 2004), maintenance and recruitment of male nurses may also be more successful by dispelling this myth and emphasize that nursing career provides opportunities to advance technical skills and includes leadership track progression. In addition, while male nurses may be more adept at care tasks that require strength such as lifting and moving patients with mobility limitations, the job should provide opportunities for problem specific solutions. For instance, engaging male nurses in solving mobility problem in the hospital such as making use of devices to move patients may be more rewarding. Such task specific interests and abilities are revealed in a study in New Zealand which found that the top ten female dominated formal care occupations include dieticians/nutritionists, nurses, midwives and occupational therapists, which typically require more personal long-term care and emotional support to the care-recipients, while male dominated occupations such as orthoptists, surgeons, physicians, and optometrists, are driven more by technical knowledge and comprise of once-off visits (Grant et al., 2004). While the study also showed that formal care occupations that were male-dominated prior to the introduction of the equal opportunities legislation became more balanced over time, occupations that were previously female-dominated remained largely female-dominated (Grant et al., 2004).

Taken together, being cognizant about sex divergent ways of problem solving and decision making in the formal care setting is critical because care provision by males may have qualitative differences compared to females. For instance, while male clinicians tend to focus on disease specific factors, offering problem-focused solutions and technical medical interventions; they are less likely to spend time assuaging patients’ feelings of worries (Bensing et al., 1993; Boerma and van den Brink-Muinen, 2000). On the other hand, female clinicians may focus on the psychoemotional and interpersonal management of the disease such as using counseling approaches (Boerma and van den Brink-Muinen, 2000), and have a greater tendency to provide continued care in the form of more frequent and more follow up consultations (Bensing et al., 1993; Jefferson et al., 2015). Instead of pushing sex equal agendas, it is likely more effective to explore further how different male and female qualities can contribute to the same formal care function.

## Discussion

In the current review, we highlight the relevance of using EP theories to understand sex differentiated preferences and competencies for formal care occupations. Sex differences as elucidated by sex-role theorists are based on the observation that many sex differences vary in magnitude across cultures and in few cases are consistent with sex-role theories (Schmitt, 2015). However, this notion has been disconfirmed by cross-cultural research observing persistent sex differences across psychological traits in personality, attitudes and cognitive abilities as predicted by EP theories (Schmitt, 2015). Furthermore, EP theories have the potential to explain why cultural universals and variations can be observed in sex differences (Pirlott and Schmitt, 2014).

While we use EP theories to elucidate the emergence of sex differences in formal care occupations, we do not think that these explanations negate the effects of socialization and culture. In addition, we acknowledge that sex differences in occupational preference may not apply to individuals who are already integrated in a sex atypical field. For instance, while males may gravitate toward things and females toward people, the EP explanations may not apply to individuals who have self-sorted into their preferred (sex atypical) occupations. Further, one should not cast judgments or dissuade individuals from pursuing a particular career path purely based on EP explanations. It may not be feasible to ensure sex parity in every occupation. However, barriers that impede females or males from advancing in the career of their choice (e.g., hiring or pay discrimination between males and females) should be systematically removed or reformed.

## Conclusion

Today, psychologists understand that pure social constructivist views are insufficient in explaining sex differences and in some instances lead to incorrect conclusions. Furthermore, evidence is clear that innate tendencies exert considerable cognitive and behavioral outcomes. Thus, giving equal weighs to EP and sociocultural theories clarifies the issues related to sex differences in formal care by enabling the understanding of sex differences as emergent phenomena of the interaction between evolved tendencies and sociocultural pressures. Ultimately, this method of examination will generate more holistic views of sex differences in formal care occupations (see Table 1 for other examples and predictions using the EP analytic approach). We propose that key decision makers within the healthcare and social care sectors work with instead of against sex differences elucidated herein and researchers to be sensitive to innate sex preferences in developing research programs. Ultimately, understanding and accepting sex differences elucidated by EP theories not only enhances our knowledge, it sheds light on how problems and research can be fine-tuned based on more precise and nuanced insights additionally informed by sociocultural theories.

**Table 1:** Examples of sex differentiated adaptive traits and the predictions for the approach, understanding, and implications of care provision.

Psychological trait	Adaptive function	Male specific functions	Female specific functions	Examples of studies	Evolutionary psychological based predictions on care provision
Emotional expression	Communicate internal emotions	Display power-oriented emotions of anger, pride, contempt to express dominance an aggressiveness	Display emotions of happiness, sadness, empathetic emotions to express affiliation	Adams et al., 2015; Güvendir, 2015; Tay, 2015	Male carers are less likely to display empathetic emotions and may be perceived to be less communicative or caring compared to their female counterparts. When assuming leadership roles, men show expressions of dominance, and aggression more readily when they encounter disagreement with colleagues. Female doctors who express happy or sad emotions more visibly would result in sex-based experience such as having patients assume that they are nurses or therapists. Females more readily display empathetic emotions when they provide care.
Relational expression	Communicate relational intent	Demonstrate dominance	Demonstrate affiliation	Taylor et al., 2000; Archer, 2004; Fromme et al., 2005	Male carers may appear less amicable, prefer leading roles, and less willing to seek opinions. Thus, male doctors will more likely to assume an authoritative stance, provide more advisory suggestions than engaging in same-level interpersonal exchange like information sharing and less likely to probe for patients’ agreeableness to their suggestions. Female carers may appear more communicative and prefer to engage other people in decision making and caregiving activities. Thus, female doctors will be more willing to ask more questions and are more comfortable to discuss personal issues or disclose upsetting news to patients. Compared to their male counterparts, female General Practitioners tend to be found in partnerships than in solo practices.
Interpersonal orientation	Social support	Kin oriented	Non-kin oriented	Rodseth et al., 1991; Foley, 1995; Taylor et al., 2000; Rudolph and Conley, 2005	Males prefer to seek help from family and relatives. Thus, they are less likely to seek social support or share their experiences with colleagues at work. Females may have a wider social support network from friends. Thus, they are more approachable and converse more readily with strangers and colleagues. They tend to share their difficulties at work.
Interpersonal social exchange	Social exchange	Hierarchical coalition, leader-follower	Reciprocal cooperation, nurturing	Baumeister and Sommer, 1997; Geary, 2010	Males may prefer to adopt leading roles or follower roles, and egalitarian social exchange may be less preferred. Male subordinates perceive duties as assigned roles and would like up tasks that enable them to display their skill sets and gain recognition. Females may prefer reciprocal forms of social exchanges where tasks are evenly shared. Female subordinates perceive duties as assistive roles and prefer tasks that enable them to display greater empathy, and provide emotional and bodily care.
Approach and avoidant tendencies	Self-enhancement, self-protection	Risk taking, competitive, accumulate resources	Cautious, cooperative, retain resources	Buss, 1989; Wilson et al., 2002; Matud, 2004	Males are more competitive and likely take on challenges in seeking prestigious jobs such as physician or surgeon. Females prefer to maintain status quo and avoid confrontational approaches. They operate on tasks such as nursing and social work.
Problem solving tendencies	Resolve physical and mental health problems	Instrumental orientation	Emotional orientation	Folkman et al., 1986; Miller and Kirsch, 1987; Davidson-Katz, 1991; Björkqvist, 1994; but see Eagly and Carli, 1981	Male clinicians may deem themselves as emotionally and interpersonally inapt and would likely refer patients to counseling services while female clinicians are more likely to spend more time and effort to discuss about patient’s emotional and interpersonal problems. Males will seek jobs that are problem- or disease-centric and have a potential solution or assigned tasks that enable them to use their skills to solve technical issues. Females will seek jobs that are patient-centric or assigned tasks relating to the caring or management of patient’s emotions.


*While we make sex distinctions, it is noteworthy that people in occupations atypical of their sex may be more similar to the opposite sex compared to people in their sex group*.

## Author Contributions

PT contributed to the conceptualization, drafting, and revision of the manuscript. YT and KT conducted the literature, and reviewed and drafted the manuscript.

## Conflict of Interest Statement

The authors declare that the research was conducted in the absence of any commercial or financial relationships that could be construed as a potential conflict of interest.
